# Low-Radiation-Dose Modified Small Bowel CT for Evaluation of Recurrent Crohn's Disease

**DOI:** 10.1155/2012/598418

**Published:** 2011-06-20

**Authors:** A. Z. Kielar, H. Tao, C. McKeever, R. H. El-Maraghi

**Affiliations:** ^1^Department of Radiology, The Ottawa Hospital, C-1, 1053 Carling Avenue, Ottawa, Ottawa, ON, Canada K1Y 4E9; ^2^Faculty of Medicine, The University of Ottawa, 451 Smyth Road, Ottawa, ON, Canada K1H 8M5; ^3^The Royal Victoria Hospital, Barrie, ON, Canada L4M 6M2

## Abstract

Crohn's disease affects any part of the GI tract, commonly the terminal ileum. To decrease radiation exposure we developed a low-radiation-dose unenhanced CT (modified small Bowel CT, MBCT) to evaluate the small bowel using hyperdense oral contrast. *Technique*. MBCT was investigated in patients with pathologically proven Crohn's disease presenting with new symptoms from recurrent inflammation or stricture. After ethics board approval, 98 consecutive patients were retrospectively evaluated. Kappa values from two independent reviewers were calculated for presence of obstruction, active inflammation versus chronic stricture, and ancillary findings. Forty-two patients underwent surgery or colonoscopy within 3 months. *Results*. Kappa was 0.84 for presence of abnormality versus a normal exam and 0.89 for differentiating active inflammation from chronic stricture. Level of agreement for presence of skip areas, abscess formation, and fistula was 0.62, 0.75, and 0.78, respectively. In the subset with “gold standard” follow-up, there was 83% agreement. *Conclusions*. MBCT is a low-radiation technique with good to very good interobserver agreement for determining presence of obstruction and degree of disease activity in patients with Crohn's disease. Further investigation is required to refine parameters of disease activity compared to CT enterography and small bowel follow through.

## 1. Introduction

Crohn's disease is an idiopathic, transmural, granulomatous disease that can affect any part of the gastrointestinal tract from the mouth to the anus, but most often affects the terminal ileum [1]. These patients usually present during their 2nd and 3rd decades with symptoms of pain, bloody diarrhea, malabsorption, weight loss, and recurrent bowel obstructions [2]. This younger patient population presents with relapsing and remitting symptoms, and up to 80% of patients with Crohn's disease require surgery at some point in the course of their disease [2]. 

Due to their recurrent symptoms, cross-sectional imaging is often requested by the treating physicians in order to identify the cause, allowing for appropriate treatment. In cases of acute flare-up, medical treatment is the best option, whereas strictures often require surgical resection. Cross-sectional imaging techniques evaluating this disease should expose the patient to the least amount of radiation while still being able to confidently and reproducibly identify the area involved, the presence of skip lesions, and whether the findings are related to acute flare-up or due to chronic structures. 

The purposes of this study were to retrospectively review interobserver agreement about MBCT findings in patients with recurrent symptoms of known Crohn's disease.

## 2. Methods

At our institution, we have developed a modified small bowel CT protocol which uses hyperdense oral contrast, 9% ioxitalamic acid (Telebrix-38, Tyco healthcare, QC, Canada), and a high noise index. A noise index of 28 is used with Auto-mA modulation on 16- or 64-slice scanners (GE Lightspeed, VCT LightSpeed (Germany)) compared to a typical noise index of 12–18 for a standard abdominopelvic CT scan. Image noise (GE Healthcare, noise index [NI]) is an operator-selected variable which alters the range of mA over which an automated tube current modulation varies during gantry rotation [3]. The combination of high NI and Auto-mA modulation reduces patient radiation dose in MBCT. 

For the MBCT protocol, patients fast for 6 hours prior to the exam. No bowel preparation or nasogastric tube insertion is needed. An oral solution of 9% Telebrix-38 (meglumine ioxitalamate; Tyco healthcare, QC, Canada) is administered compared to the 3% used as oral preparation in most standard CT scans of the abdomen and pelvis. This solution has a high attenuation (500–600 HU), increasing contrast resolution on this unenhanced CT protocol. Hounsfield units in excess of this would cause beam hardening artifacts. The patient drinks 300 mL aliquots of the oral contrast every 15 minutes over 60 minutes, for a total volume of 1200 mL. The isotonic nature of the 9% oral contrast gives better distension of the bowel lumen compared to water or other lower osmolal solutions, since there is physiologically less absorption of this isotonic solution by the small bowel. This allows more consistent distension of the small bowel, even in patients without moderate or high-grade small bowel obstruction.

Timing of the CT scan is determined on scout images, since the hyperdense oral contrast is readily visualized in the ascending colon, indicating complete small bowel opacification. 

Other parameters include 120 kV, mA range of 100 to 240 mA, a 0.5 sec gantry rotation, and a pitch of 1, with slice reconstruction of 0.5–1.25 mm, depending on the scanner used. Prone and supine scanning ensures opacification of all parts of the small bowel, but at the discretion of the radiologist, the prone scan can be omitted, particularly in follow-up studies. Images are viewed at a window of 800–900 HU and level of 200–300 HU.

Reconstructed thin slices are postprocessed to produce maximum intensity projections (MIP), multiplanar reformats (MPR), and virtual endoscopy images, which have been shown to increase diagnostic confidence [4, 5]. Once reformatted on a 3D workstation and viewed as thicker cuts, the resultant images depict density differences between the dense oral contrast in lumen of the small bowel, the grey wall of the bowel, and the dark fat surrounding the bowel as well as the dark grey appearing vasculature in the small bowel mesentery. On average, reconstructions take less than 5 minutes but may require up to 20 minutes, depending on the case's complexity.

We performed a HIPPA compliant, REB-approved, retrospective review of 98 consecutive patients with previously diagnosed Crohn's disease who presented for investigation of new symptoms which were suspected to be due to either inflammation or chronic stricture. The diagnosis of Crohn's disease was pathologically proven previously, either by surgery or colonoscopy with ileal intubation and/or endoscopy with biopsies. 

Each modified small bowel CT image sets were retrospectively reviewed by two radiologists in a blinded fashion: the prior reports were not available to them and prior images were not accessed. A standardized research form was filled out which qualitatively evaluated the adequacy of bowel distension, the degree of confidence of the final diagnosis, whether or not the study was normal or abnormal, which bowel segments were involved and whether the findings were consistent with acute inflammation or chronic stricture, and existence of ancillary findings. Ancillary findings included presence of abscesses, fistula formation, and skip lesions; each of these variables was independently documented. 

Statistical analysis software was used to calculate the kappa scores. The kappa statistics for interobserver agreement for binary CT signs were interpreted using the following scale: fair agreement, 0.21–0.40; moderate agreement, 0.41–0.60; substantial agreement, 0.61–0.80; and almost perfect agreement, 0.81–1.0 [6].

## 3. Results

There were 49 men and 49 women with average age of 44 years old (range 22–88 years old). All had previously biopsy-proved Crohn's disease and were referred by a gastroenterologist or surgeon with symptoms referable to their disease (either due to acute flare-up or chronic stricture). 

The qualitative analysis of the adequacy of bowel distension revealed that 74/98 cases were deemed to be of good quality and 24 of average quality. None were considered to be of poor quality by either Reader. The degree of distal small bowel distension was the key factor in determining adequacy of the study however distension of the rest of the proximal small bowel loops was also considered for determining adequacy of the study as well as visualization of the subtending mesentery within the intra-abdominal fat. 

The level of confidence regarding the overall diagnostic outcomes was assumed to be high in 69/98 cases for Reader 1, intermediate in 25 cases, and low in 4 cases. For Reader 2 (who had more experience with this technique), the level of confidence regarding the findings was high in 93/98 cases, intermediate in 5 cases, and low in 1 case. 

A kappa score for interobserver variability for overall determination of normal versus abnormal study was 0.84. 28 studies were considered normal by Reader 1 and 38 were normal by Reader 2, though in a few cases, Reader 1 indicated abnormal study due to presence of significant postoperative changes with mild strictures which Reader 2 acknowledged to be “normal” for this patient since the area of narrowing was not causing moderate or high-grade obstruction.

**Figure 1:**
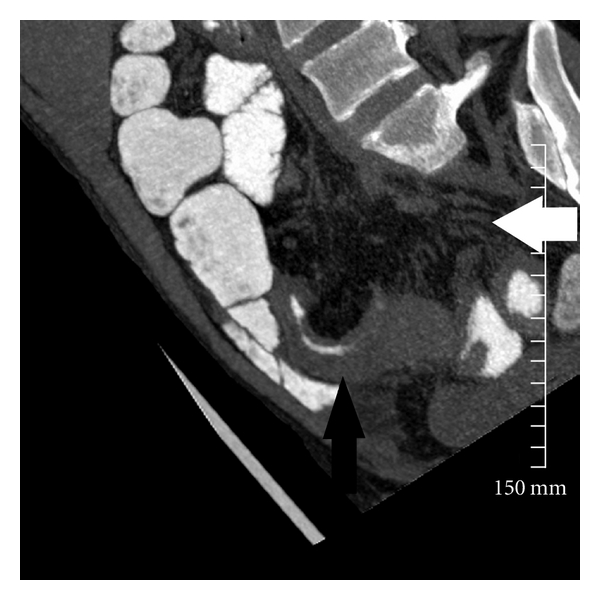
38-year-old patient with Crohn's disease with 8 mm thick slab reconstruction in coronal-oblique plane. There is mural thickening of the ileum (black arrows), increased mesenteric fat attenuation, and prominence of the vasa recta (white arrowhead), all in keeping with active disease superimposed on chronic changes.

**Figure 2:**
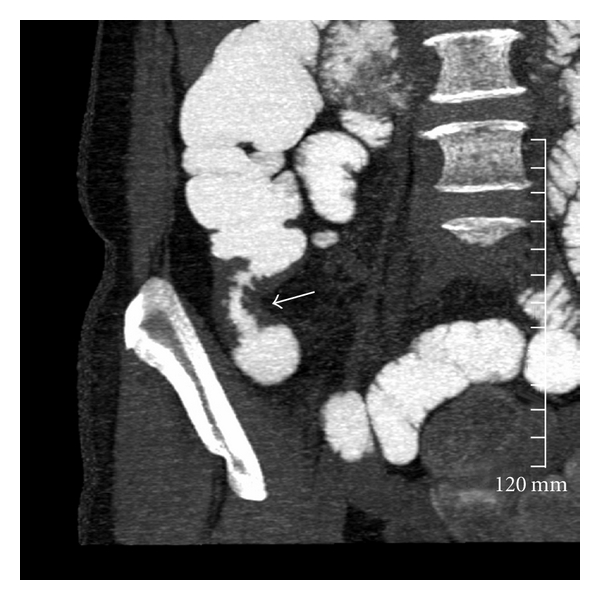
Coronal thick-slab-reconstructed image in a patient with documented Crohn's disease with a chronic stricture causing low-grade obstruction (white arrow).

In the abnormal studies, the kappa value for active inflammation versus chronic stricture was 0.89 (Figures 1 and 2). The remaining kappa values for bowel segments affected, presence of skip lesions, presence of abscess, and presence of fistula are presented in Table 1. The segment of bowel involved was separately recorded, and the results are depicted in Table 2. The values for kappa ranged from 0.53 to 0.78 for different segments. 

**Figure 3:**
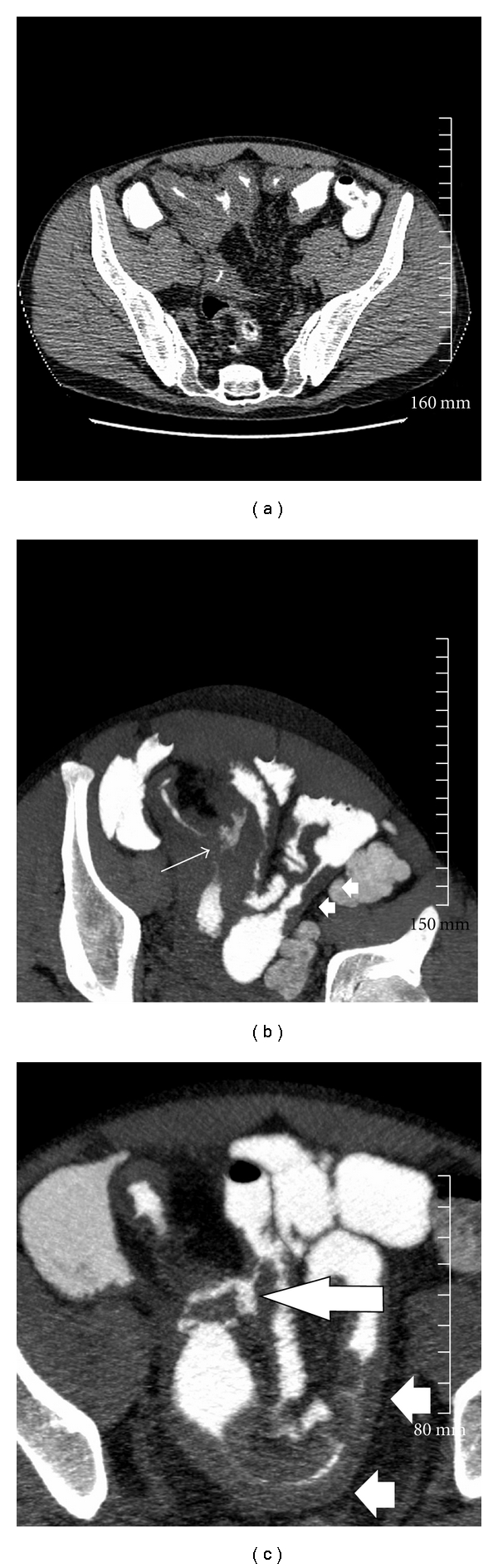
(a) 42-year-old male with Crohn's disease. Raw supine, axial images acquired at 1.25 mm slices prior to reconstruction on the 3D workstation. (b) The 7 mm thick, axial-reconstructed MBCT oblique-axial reformats demonstrate an enteroenteric fistula (arrow). There is also a skip lesion in small bowel on the left side of the pelvis (white arrows heads). (c) Follow-up imaging 1 year later demonstrates that this enteroenteric fistulous connection persists (arrow). On the current study, the skip lesion seen previously demonstrates increasing thickness indicating worsening of disease (arrowheads).

Reader 1 detected 21 patients who had skip areas compared to 29 cases for Reader 2. 8 abscesses were detected by Reader 1 compared to 5 by Reader 2, though there were 2 areas of possible phlegmon identified by Reader 2 which were not documented as frank abscesses. 9 cases of fistula were detected by Reader 1 compared to 11 by Reader 2 (Figures 3(a) and 3(b)). 

Incidental findings were also retrospectively recorded from the original radiology reports. There were 3 patients with incidentally discovered renal stones, 2 with calcified gallstones, 2 abdominal aortic aneurysms, 4 with significant fatty infiltration of the liver, and other incidental findings, such as 2 cases of large fibroids and 2 uncomplicated inguinal hernias. 

Fifty-four of 98 patients underwent follow-up studies within 4 months which were considered to be “gold standards.” 32 patients had surgery or colonoscopy with ileal intubation and 24 patients had followup cross-sectional imaging. 46 of 54 (85%) of cases that had “gold standard” follow-up were in agreement with MBCT. In 7 of the 8 cases of disagreement, the gold standard revealed a different and clinically significant finding from the MBCT. This included 2 false positive findings, 5 false-negative finding, and 1 other missed diagnosis. 

In order to reduce radiation further in patients who had previously underwent cross-sectional imaging and had a known area of fistula or abscess, only supine scanning was obtained. Supine imaging was chosen rather than prone since CT enterography and standard CECT are performed in this manner and, thus, it was though that same patient positioning would reduce differences in reporting due to bowel loop position. Radiation exposure was estimated from the recorded dose length product (DLP). The average DLP, a value generated by the CT console was 391 mGycm and range was (187–908 mGycm). Only supine imaging was performed in 7 cases. Using the formula for estimated radiation exposure E = DLP × 0.015 (abdominal conversion factor) [7], this is equal to approximately 5.8 mSv for the combination of either prone and supine imaging or only supine image acquisitions.

## 4. Discussion

There are many options for imaging Crohn's disease. Small bowel follow through and small bowel enteroclysis were used for many decades however due to overlapping bowel loops, abnormalities such as skip areas, or focal low-grade stricture could be missed [8]. 

 CT has become a common way of reproducibly evaluating the small bowel, especially since the advent of CT enterography (CTE) [9, 10]. CTE is a cross-sectional imaging modality which involve spositive intravenous contrast and negative oral contrast. Various negative oral contrasts have been evaluated including Volumen (Volumen; E-Z-EM, Westbury, NY, USA, 0.1% w/v ultra-low-dose barium with sorbitol, a nonabsorbable sugar alcohol), water, milk, and polyethylene glycol [9–12], but obtaining adequate distension is often challenging, particularly if there is no acute obstruction.

 Due to the relatively young age of many patients with Crohn's disease, radiation exposure is a concern. Radiation dose from MBCT is lower than conventional CTE and small bowel follow through (SBFT). Barium procedures such as SBFT and enteroclysis have been reported to have effective doses of 13.68 ± 6.85 mSv and 13.99 ± 7.57 mSv, respectively [8]. CTE mean effective doses have been reported at 9.58 mSv, with additional 1.13 mSv for the placement of the nasojejunal tube in CT enteroclysis [9]. In comparison, radiation dose from a supine acquisition with the above parameters has a dose length product (DLP) of approximately 150 mGycm, which equates to only 2.55 mSv. It does not use intravenous dye and is, therefore, safer for patients and less expensive than CTE or standard contrast-enhanced CT.

MRI is starting to become more commonly used for small bowel imaging, but MR enterography usually requires the use of intravenous contrast which is associated with a low but real risk of allergic reaction. Additionally, scan times are in the range of 30–45 minutes compared to only a few seconds when performing CT and antiperistaltic agents are usually required [13–16].

Ultrasound has demonstrated high interobserver agreement in a meta-analysis of prospective studies performed by Horsthuis et al. [17], however, evaluation of the bowel using ultrasound can still be negatively affected by overlapping, air-filled bowel loops. Additionally, detection and characterization of complex enteroenteric fistulas can be difficult partly due to heavy reliance on completeness of bowel evaluation with inter- and intra-observer variations. 

One of the key features of the MBCT protocol is the ability to change the NI before starting the CT scan. Due to the high intrinsic contrast resolution related to the presence of the 9% Telebrix-38, the noise index can be increased without losing the contrast resolution between the hyperdense bowel lumen, the grey wall, and the surrounding dark fat. 

Complications of Crohn's disease include acute or subacute bowel obstruction. This may be due to either acute inflammation or chronic stricture formation. Differentiation of these two entities can be difficult but is important because management varies according to cause. For example, acute inflammation is usually treated medically with anti-inflammatory agents, steroids, ASA-derivative agents, and immune modulators [18] whereas chronic strictures may require surgical resection. In our patient population who had an abnormal finding on MBCT, the kappa value for active inflammation (with or without underlying chronic components) versus predominantly chronic strictures was 0.89 which is considered “almost perfect.” Active inflammation in an area of chronic disease was considered as “active” disease for the purposes of this study even if there were likely components of both. Imaging features suggestive of active inflammation included prominence of the gray subtending mesentery, haziness in the fat around the bowel wall, and ill definition between the edge of the wall and the adjacent fat. Chronic stricture typically demonstrated a well-defined boarder between the wall and the fat with less prominence of the underlying mesenteric vessels.

The kappa values for segment of bowel involved ranged from 0.53 to 0.78. Part of the lower kappa results were that certain bowel segments were infrequently involved (duodenum). Also, the definition of neoterminal ileum and distal ileum was controversial, particularly in postoperative patients and there was some interobserver disagreement in cases, though on review of the images, the readers were indicating the same area of interest in many instances. 

In the subgroup of patients who underwent “gold standard” followup, there were 8 discrepant cases. In the first case, MBCT described active Crohn's disease of the terminal ileum. At surgery, this was found to be a Meckel's diverticulitis. In the second false-positive MBCT case, active Crohn's disease of the terminal ileum was found to represent a stricture 3 months later, however, the patient had interval medical therapy and, thus, this may not be a truly discrepant case, rather, the active inflammation may have changed to scarring and fibrosis in the interim. 3 cases were described as being normal on the original MBCT reports. In one case, colonoscopy with ileal intubation 3.5 months later demonstrated a single aphthous ulcer in the terminal ileum. In another case which underwent ileoscopy 3 months later, a small ulcer was found 5 cm from the stoma in a patient with prior colectomy. In the third case, mild inflammation was demonstrated on colonoscopy at the neoterminal ileum a few days after “normal” MBCT. In the group of false-negative MBCT, in one case, no leak was detected by MBCT but 2 months later, the patient was taken to the operating theatre because of persistent fevers and pain, and a small leak at the anastomosis was found and repaired. In another case, MBCT was reported as normal but colonoscopy identified Crohn's colitis. In retrospect, MBCT did demonstrate this finding, though this study is geared to opacify and evaluate the small bowel rather than focus on the colon. In the final discrepant case, MBCT correctly identified active Crohn's disease of the terminal ileum but did not detect a pancreatic mass. A follow-up CECT 3 months later showed the ill-defined, hypoenhancing pancreatic mass from which the patient later died. 

Since there is no intravenous contrast used for this MBCT, solid organs cannot be adequately evaluated. This type of CT protocol should not be used in older patients with chronic anemia or nonspecific abdominal symptoms. It should also not be used in cases of suspected malignancy. Additionally, it should not be used in patient with acute GI bleed, since there is no intravenous contrast used and the hyperdense oral contrast would obscure subsequently injected contrast into the lumen of the bowel. 

One of the limitations of this study is its retrospective nature, and there was no gold-standard comparison available for all the cases. Thus, the true sensitivity, specificity, and accuracy of MBCT could not be calculated. Most patients diagnosed with acute inflammation are treated medically and, thus, there is no consistent surgical intervention or pathologic diagnosis. We did not divide acute or chronic disease as a separate category, but simply categorized them as acute inflammation. However, of the patients who did undergo other imaging or intervention, there was 85% agreement as discussed above. 

We also did not quantify the degree of distension of the bowel. This is technically difficult, particularly in patients who have had prior bowel resection and in patients with strictures causing partial obstruction. A more quantitative determination of the degree of bowel distension could be attempted in future studies.

## 5. Conclusions

MBCT is a low-radiation technique used to determine the involved bowel segments, presence of obstruction, and degree of disease activity in patients with known Crohn's disease. For this indication, MBCT is a highly reproducible imaging technique with good and very good kappa scores for evaluating parameters such as presence of active disease versus stricture, the presence of abscess, strictures, and skip lesions. 

Further study with prospective trials should be conducted, with direct comparison to CTE and MRE with colonoscopic or surgical comparison. 

## Figures and Tables

**Table 1:** Kappa scores for MBCT results in patients with followup of pathologically proven Crohn's disease.

Characteristic	Kappa value
Normal versus abnormal MBCT (overall)	0.84
Active inflammation versus stricture	0.89
Bowel segments involved	0.53–0.78
Presence of skip areas	0.62
Presence of abscess	0.75
Presence of fistula	0.78

Poor agreement ≤ 0.20.

Fair agreement = 0.20 to 0.40.

Moderate agreement = 0.40 to 0.60.

Substantial agreement = 0.60 to 0.80.

Almost perfect agreement = 0.80 to 1.00.

**Table 2:** Bowel segment affected by acute or chronic crohn's disease.

	Reader 1	Reader 2	Kappa
Duodenum	4	5	0.74
Jejunum	20	28	0.53
Ileum	51	51	0.78
Terminal ileum	20	28	0.70
Colon	28	35	0.62
